# A Three-Compartment Pharmacokinetic Model to Predict the Interstitial Concentration of Talaporfin Sodium in the Myocardium for Photodynamic Therapy: A Method Combining Measured Fluorescence and Analysis of the Compartmental Origin of the Fluorescence

**DOI:** 10.3390/bioengineering6010001

**Published:** 2018-12-21

**Authors:** Yuko Uno, Emiyu Ogawa, Eitaro Aiyoshi, Tsunenori Arai

**Affiliations:** 1 School of Fundamental Science and Technology, Graduate School of Science and Technology, Keio University, 3-14-1, Hiyoshi, Kohoku-ku, Yokohama City, Kanagawa 223-8522, Japan; tsunearai@appi.keio.ac.jp; 2 School of Allied Health Science, Kitasato University, Kanagawa 252-0373, Japan; e.ogawa@kitasato-u.ac.jp; 3 The Institute of Statistical Mathematics, Tokyo 190-0014, Japan; aiyoshi0823@gmail.com; 4 Department of Applied Physics and Physico-Informatics, Faculty of Science and Technology, Keio University, Kanagawa 223-8522, Japan

**Keywords:** talaporfin sodium, pharmacokinetics, three-compartment model, interstitial space, myocardial fluorescence

## Abstract

To evaluate the effectiveness of photodynamic therapy occurring in the interstitial space of the myocardium, we estimated the interstitial concentration of talaporfin sodium in the canine myocardium by constructing a three-compartment pharmacokinetic model based on measured changes in talaporfin sodium plasma concentration and myocardial fluorescence. Differential rate equations of talaporfin sodium concentration in the plasma, interstitial space, and cell compartment were developed with individual compartment volume, concentration, and rate constants. Using measured volume ratios based on histological examinations, we defined that the myocardial fluorescence consisted of the linear addition of fluorescence generated from these three compartments. The rate constants were obtained by fitting to minimize the sum of the squared errors between the measured talaporfin sodium concentrations and the calculated concentrations divided by the number of data points using the conjugate gradient method in MATLAB. We confirmed that this fitting operation may be appropriate, because a coefficient of determination between the measured talaporfin sodium changes and the calculated concentrations using our equations was 0.99. Consequently, to estimate the interstitial concentration in the canine myocardium, we propose a three-compartment pharmacokinetic model construction methodology using measured changes in talaporfin sodium plasma concentration and changes in myocardial fluorescence.

## 1. Introduction

We have studied the application of an extracellular photosensitization reaction (PR) using talaporfin sodium to realize a low-temperature-elevation myocardial arrhythmia ablation method [1,2,3,4]. We developed a three-compartment pharmacokinetic model to estimate the interstitial concentration of talaporfin sodium in canines using measured talaporfin sodium myocardial fluorescence. Talaporfin sodium (Meiji Seika Pharma Co. Ltd., Tokyo, Japan), known as a clinical photosensitizer “Laserphyrin” in Japan, is a second-generation photosensitizer and has an absorbance peak at 664 nm in the Q band [5]. This method is based on a PR with a high concentration of talaporfin sodium in the myocardial interstitial space, because of a short drug-light interval [1,2,3,4]. With singlet oxygen production outside myocardial cell, an immediate electrical conduction block can be realized within a few minutes in animal models [6]. In this scheme, the myocardial interstitial space is our targeted PR region, and concentration changes of talaporfin sodium in the interstitial space are needed to obtain the maximal interaction efficacy. In terms of our application, talaporfin sodium in the interstitial space plays a major role in the therapeutic effect. The therapeutic effect would be successful when the talaporfin sodium concentration in the interstitial space is within the effective therapeutic concentration range. We can determine the start and end timing of the therapeutic effect after intravenous injection of talaporfin sodium in our application using the estimated talaporfin sodium concentration in the interstitial space. In addition, it is generally hard to measure talaporfin sodium concentration in the interstitial space in vivo. The final result of the therapeutic effect would be judged from animal experiments, but it is also important to be able to theoretically explain it by describing a three-compartment pharmacokinetic model. Therefore, the constructed three-compartment pharmacokinetic model can provide very useful information. We intended to predict the interstitial concentration of talaporfin sodium to evaluate the effectiveness of this application, moreover for interstitial photodynamic therapy (PDT), such as infectious disease treatment [7]. After intravenous injection of talaporfin sodium, it diffuses from the plasma into the interstitial space and from there to cell.

There are many reports of photosensitizer pharmacokinetics using a two-compartment pharmacokinetic model of plasma and tissue [8,9,10]. However, there are no reports describing estimated talaporfin sodium concentration in the interstitial space using a pharmacokinetic model. Therefore, we propose a methodology to construct a three-compartment pharmacokinetic model consisting of the plasma, interstitial space, and cell, and using measured fluorescence to estimate the talaporfin sodium interstitial concentration. The cell compartment was set as the functional tissue, which is similar to the conventional two-compartment model. To construct the three-compartment model, we performed conventional measurement of talaporfin sodium concentration in canine plasma, and novel measurement of fluorescence changes in canine myocardium. 

Using fluorescence measurements, we could construct a three-compartment pharmacokinetic model in an easy and safe way. This is in contrast to the few reports that describe a three-compartment pharmacokinetic model using radioactive substances to estimate the interstitial concentration [11]. We constructed a three-compartment pharmacokinetic model estimating talaporfin sodium concentration dynamics in the canine myocardial interstitial space. This model can be useful, not only for our application, but also for PDT with high concentrations of photosensitizer in the interstitial space, such as for treatment of infectious disease. Therefore, to estimate canine myocardial interstitial concentration, we present the construction of a three-compartment pharmacokinetic model using measured talaporfin sodium plasma concentration changes and myocardial fluorescence changes with consideration of the origin of the fluorescence from the compartments with measured volume ratios based on histological examinations.

## 2. Materials and Methods 

### 2.1. Measurement of Plasma Concentration and Myocardial Fluorescence of Talaporfin Sodium in Canines

Typically, the speed of talaporfin sodium metabolism differs greatly among animal species. If the speed of metabolism is different, the talaporfin sodium pharmacokinetics and the therapeutic effects are different. Normally, for cardiovascular studies, pigs are used as a mid-size animal model. However, the speed of metabolism in pigs is about 50 times higher than that of humans because albumin structure differs between pigs and humans [12]. Therefore, we considered that pigs were not a suitable animal for our experimental model. We chose the canine as a better animal model because the metabolism of canines is closer to that of humans among mid-size animals. The speed of metabolism in canines is about five times faster than that of humans [13]. In addition, the diazepam-binding site, which is an important factor to evaluate the therapeutic efficacy in the interstitial PDT, only exists in canine and human serum albumin among canines, humans, pigs, and cows [14,15]. From the viewpoint of serum albumin species, the interstitial PDT efficacy (described in Section 1) might be similar in canines and humans.

**Figure 1:**
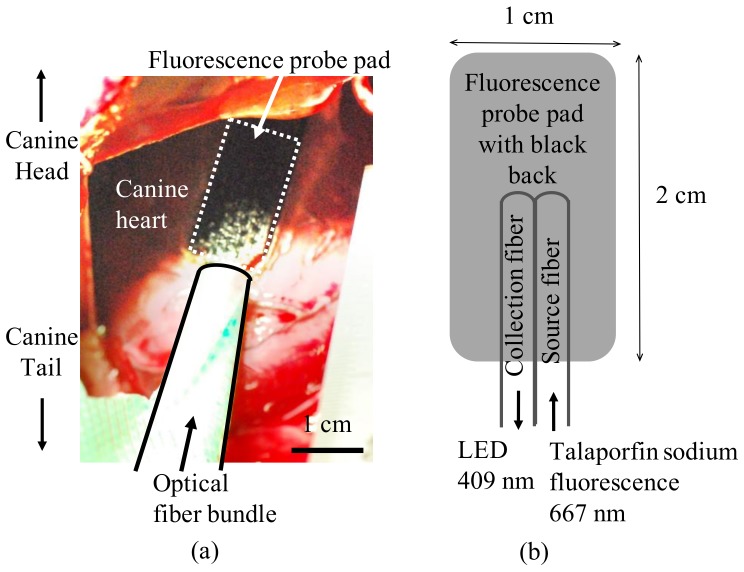
Fluorescence-sensing probe pad installation on an open-chested beagle heart. (**a**) Image of the fluorescence-sensing probe pad installation; (**b**) structure of the fluorescence-sensing probe pad.

An intravenous bolus of talaporfin sodium (2.5 mg/kg) was administrated into a superficial femoral vein of a beagle canine (male, 9 months old, weighing 9.3 kg). Using a pre-measured calibration curve between talaporfin sodium absorption and concentration, the concentration in the plasma (*M*_plasma_) of blood samples was obtained using the visible absorption spectrum measured using a microvolume spectrophotometer (Titertek-Berthold, Pforzheim, Germany) immediately before, and 5, 10, 15, 20, 30, 40, 50, 60, 70, 75, and 85 min after injection. The blood sample volume was 1 mL. Because we wanted to know the interstitial myocardial concentration of talaporfin sodium, its fluorescence in the canine myocardium was measured using a fluorescence-sensing probe, as described previously [16]. The fluorescence-sensing probe pad shown in Figure 1 was attached to the open-chested beagle canine heart while avoiding the major coronary arteries.

The optical detection system used to measure the myocardial fluorescence was also described previously [16]. The fluorescence was measured at a wavelength of 667 nm after excitation at 409 nm. The relative intensity of the myocardial surface fluorescence (*M*_myo_) was measured immediately before, as well as 10 and 40 min after injection. The fluorescence represents the relative value of concentration; therefore, the measured fluorescence was translated to the absolute myocardial concentration (*C’*_myo_) using a conversion constant (*R*_myo_) as described in Equation (1).

(1) $$C_{\mathrm{myo}}^{\prime }=R_{\mathrm{myo}}\cdot M_{\mathrm{myo}}$$

### 2.2. Determination of the Components of Talaparfin Sodium Myocardial Fluorescence Based on Histological Examinations

**Figure 2:**
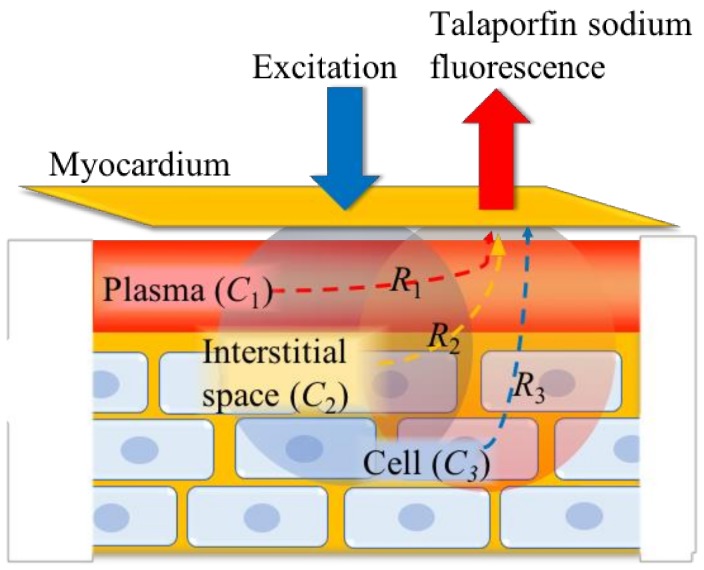
Origin of the measured talaporfin sodium concentration changes in the myocardium with volume ratios, *R*_1_, *R*_2_, and *R*_3_, which indicate the volume ratios of the plasma, interstitial space, and cell in the measured myocardial fluorescence, respectively.

The measured myocardial talaporfin sodium fluorescence provides concentration information of not only the interstitial space but also of plasma and cell, as shown in Figure 2. 

We then defined that the myocardial fluorescence consisted of the linear addition of fluorescence generated from three compartments, plasma, interstitial space, and cell, using volume ratios based on histological examinations. We obtained a volume ratio of plasma, interstitial space, and cell compartments in the fluorescence by microscopic histological examinations of the canine myocardium. Myocardial samples from the dog were extracted, fixed with a 10% formalin solution, and embedded in paraffin. The paraffin-embedded specimens were sectioned at a thickness of 6 μm and separately stained with alpha smooth-muscle-actin and hematoxylin-eosin (HE) individually. These stained samples were observed using a microscope (FSX100, Olympus Co., Tokyo, Japan) at 60 × magnification. To fix investigation depth of these samples, we determined the fluorescence sampling depth. This depth of the thin myocardial section used was approximately half the penetration depth of the myocardium for 667 nm light, which was 2.4 mm, because the fluorescence sampling was governed by the round-trip of excitation and fluorescent light.

The total blood vessel area in the cross-section of the myocardial sample within the investigation depth was calculated using the inner luminal area of the alpha smooth-muscle-actin-stained vessel walls. The blood vessel area ratio was calculated by dividing this area by the myocardial cross-section within the investigation depth. Fifty-five percent of the blood vessel area ratio was used as the plasma area ratio (*R*_1_) in this cross-section, assuming a hematocrit level of 0.45 [17]. The number of cells in the cross-section was counted by binarized HE-stained cell nucleus imaging using ImageJ 1.51 (National Institute of Health, Bethesda, MD, USA). The total cell area in the cross-section was obtained by multiplying a cell unit area, assumed to be a sphere of 18.5 μm [18,19], by the number of cells. The cell area ratio (*R*_3_) in the cross-section was obtained by dividing the total cell area by the area of the cross-section. To determine the interstitial area ratio (*R*_2_) of the cross-section, fat and collagen fibers, 11.7 vol% [20] and 3.9 vol% [21], respectively, were extracted from the entirety.

### 2.3. Determination of the Compartment Volumes in the Three-Compartment Model

Since there were several optimized parameters in our proposed three-compartment model, unlike in the conventional two-compartment model, we thought that it would be better to reduce the number of optimized parameters under our model construction policy. We thought that the accuracy of the model would decrease if compartment volumes were also optimized. We, therefore, used a fixed value for the compartment volume, not a value estimated from the model. The plasma compartment volume (*V*_1_) was obtained physiologically. The blood volume in a canine constitutes 7.7% of the total body weight [22]. The plasma compartment volume was 55% of that, as described in Section 2.2. The interstitial compartment volume (*V*_2_) and the cell compartment volume (*V*_3_) were obtained functionally. Glucose and talaporfin sodium are both water-soluble and close in molecular weight; therefore, we assumed that the pharmacokinetics for both would be similar, but of course it is not identical. The tissue compartment volume using a two-compartment model of glucose for canine was 1221 mL [23], after correcting for the canine weight of 9.3 kg. The corrected volume was considered to be the sum of the interstitial space and cell compartment volume in the constructed three-compartment model. These values were obtained using the measured interstitial and cell area ratios described in Section 2.2.

### 2.4. Three-Compartment Mathematical Modeling of Pharmacokinetics

**Figure 3:**
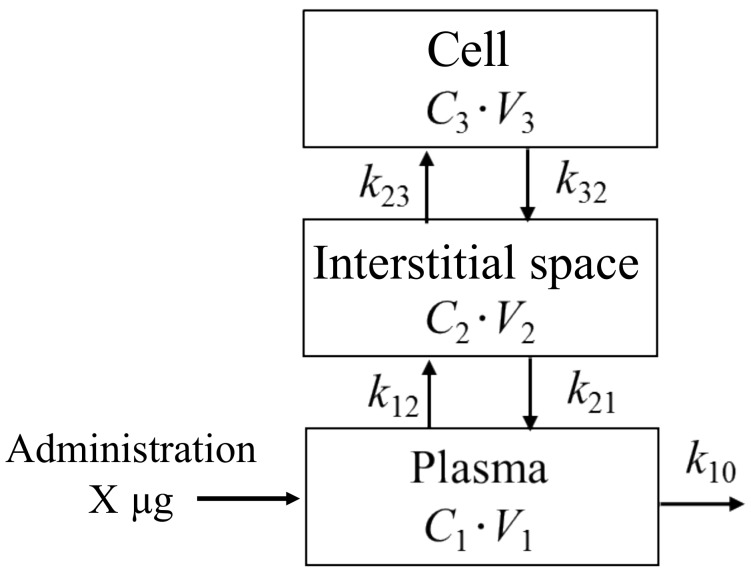
Schematic of the proposed three-compartment pharmacokinetic model.

Figure 3 shows a schematic diagram of the proposed three-compartment pharmacokinetic model consisting of the plasma, interstitial space, and cell compartments. The subscript 0 denotes space outside of the model. The subscripts 1, 2, and 3 denote the plasma, interstitial space, and cell compartments, respectively. The drug quantity can be described by a series of differential Equations (2)–(4).

(2) $$V_{1}\frac{dC_{1}}{dt}=-(k_{10}+k_{12})V_{1}C_{1}+k_{21}V_{2}C_{2}$$

(3) $$V_{2}\frac{dC_{2}}{dt}=k_{12}V_{1}C_{1}-(k_{21}+k_{23})V_{2}C_{2}+k_{32}V_{3}C_{3}$$

(4) $$V_{3}\frac{dC_{3}}{dt}=k_{23}V_{2}C_{2}-k_{32}V_{3}C_{3}$$

In these equations, *V*_i_ and *C*_i_ indicate each compartment volume and concentration, respectively (i = 1, 2, and 3). The excretion rate constant from the plasma compartment and the rate constants between each compartment are written as *k*_10_, *k*_12_, *k*_21_, *k*_23_, *k*_32_, as shown in Figure 3.

The concentration in the myocardium ($C_{\mathrm{myo}}$) can be described by the following Equation (5) (see Appendix A)

(5) $$C_{\mathrm{myo}}=R_{1}C_{1}+R_{2}C_{2}+R_{3}C_{3}$$

where, *R*_1_, *R*_2_, *R*_3_, indicate the volume ratios of the plasma, interstitial space, and cell for the measured myocardial fluorescence, respectively. Our idea is that the fluorescence in the myocardium consists of the linear addition of fluorescence generated from three compartments using the volume ratios. Also, the fluorescence represents the relative value of the concentration. Therefore, we can describe the concentration in the myocardium as the linear addition of the concentration of the three compartments using the volume ratios.

**Figure 4:**
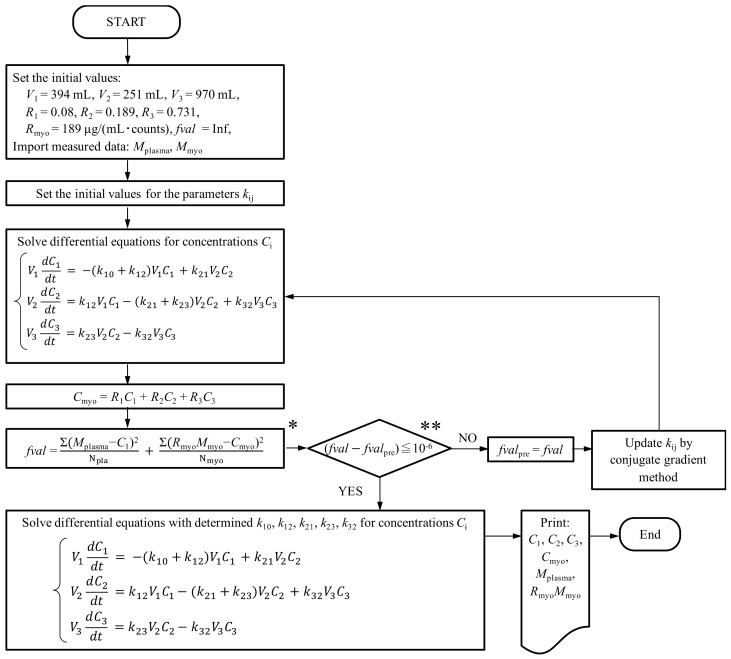
Diagram of the proposed optimization procedure. (* N_pla_ and N_myo_ represent the number of data points for the plasma concentration and myocardial fluorescence, respectively. ** *fval*_pre_ represents the previously calculated value of *fval*).

In the proposed optimization procedure to identify *k*, as shown in Figure 4, *V*_1_, *V*_2_, *V*_3_, *R*_1_, *R*_2_, and *R*_3_ are found based on the histological examinations described in Section 2.2 and 2.3. The initial concentration of the plasma compartment [*C*_1_(0)] was obtained by dividing the initial talaporfin sodium administration of X μg by *V*_1_, and the initial concentration of the interstitial compartment [*C*_2_(0)] and the cell compartment [*C*_3_(0)] were set to 0 μg/mL. A conversion constant (*R*_myo_: *C*′_myo_ = *R*_myo_ · *M*_myo_, see Section 2.1) to obtain the absolute talaporfin sodium concentration from the measured myocardial fluorescence was determined to match the initial values of the myocardial measured concentration data [*C*′_myo_(0)] and the myocardial concentration [*C*_myo_(0)] calculated from Equation (5). The rate constants, *k*_10_, *k*_12_, *k*_21_, *k*_23_, *k*_32_, were optimized to minimize *fval*, the sum of the squared errors between the measured plasma concentration (*M*_pla_) and calculated plasma concentration (*C*_1_), and the measured myocardial concentration (*C*′_myo_) and calculated myocardial concentration (*C*_myo_) divided by the number of data points using the conjugate gradient method with the solver “fmincon” in MATLAB R2016a (Mathworks, Natick, MA, USA). 

## 3. Results

### 3.1. Determination of Volume Ratios (R_1_, R_2_, and R_3_) and Compartment Volumes (V_1_, V_2_, and V_3_)

The values of the volume ratios *R*_1_, *R*_2_, and *R*_3_ were obtained by the method described in Section 2.2. The blood vessel and plasma area ratios in the myocardium cross-section were 12.3 ± 2.1% and 6.77%, respectively. The cell area ratio in the myocardium cross-section was 61.7 ± 7.7%. The interstitial area ratio was calculated as 15.9%. The volume ratios of plasma (*R*_1_), interstitial space (*R*_2_), and cell (*R*_3_) to the myocardial fluorescence were 0.08, 0.189, and 0.731, respectively, with the sum fixed at 1. Finally, we obtained the following Equation (6).

(6) $$C_{\mathrm{myo}}=0.08C_{1}+0.189C_{2}+0.731C_{3}$$

The values of the compartment volumes *V*_1_, *V*_2_, and *V*_3_ were obtained by the method described in Section 2.3. The plasma compartment volume was calculated physiologically to be 394 mL (*V*_1_). The interstitial compartment volume of 251 mL (*V*_2_) and the cell compartment volume of 970 mL (*V*_3_) were obtained functionally. A conversion constant (*R*_myo_) was set to be 189 μg/(mL·counts) described in Section 2.4 and Appendix A. Finally, we obtained the following Equation (7).

(7) $$C_{\mathrm{myo}}^{\prime }=189\cdot M_{\mathrm{myo}}$$

### 3.2. Construction of the Three-Compartment Model Using the Measured Plasma Concentration and Myocardial Fluorescence

**Figure 5:**
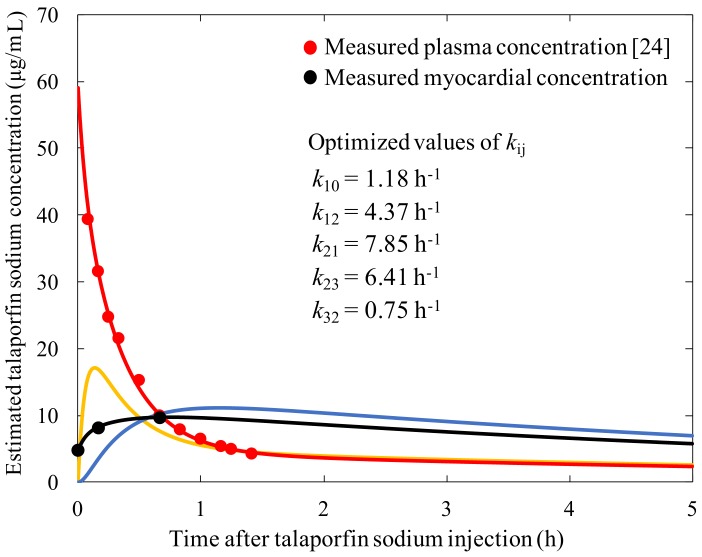
Estimated talaporfin sodium concentration time history using the three-compartment model. (Red line: estimated concentration in the plasma compartment; orange line: estimated concentration in the interstitial space compartment; blue line: estimated concentration in the cell compartment; black line: estimated concentration in the myocardium; the red plots: measured plasma concentration [24]; and black plots: measured myocardial concentration.).

The estimated talaporfin sodium concentration changes in each compartment and in the myocardium are shown in Figure 5. The measured talaporfin sodium concentrations are also plotted in Figure 5. The measured plasma concentration data were obtained from the literature [24].

Fitting to minimize *fval* as described in 2.4, the rate constants in the three-compartment model were obtained (Figure 5). We confirmed that the fitting operation was appropriate because the coefficient of determination between the measured data and calculated concentration using our equations was 0.99. The estimated talaporfin sodium concentration peak in the interstitial compartment was observed at 8 min after injection.

## 4. Discussion

### 4.1. Methodology of the Constructed Three-Compartment Model for Talaporfin Sodium Using the Myocardial Fluorescence Time History and Volume Ratios Measured from Histological Examinations

We developed a three-compartment model using measured talaporfin sodium fluorescence from canine myocardium and measured volume ratios. There have been many reports on the pharmacokinetics of photosensitizers, but few have looked at talaporfin sodium [25]. One such study using talaporfin sodium used a two-compartment model in cancer patients [25]. PDT using talaporfin sodium has been conventionally used as a minimally invasive cancer therapy with selective photosensitizer accumulation in tumor cell [26,27]. For cancer, PR occurs almost entirely in cell. Therefore, a two-compartment model is sufficient to evaluate the therapeutic effectiveness of PDT for cancer. However, as mentioned in Section 1, in our proposed interstitial PR scheme for myocardial ablation or infectious disease, the concentration in the interstitial space is temporarily high shortly after drug administration. It is, therefore, necessary to construct a three-compartment model to determine the interstitial concentration changes.

The constructed model utilizes not only changes in talaporfin sodium plasma concentration but also changes in talaporfin sodium fluorescence at the myocardial surface to determine the rate constants in the equations. The measured myocardial fluorescence (*M*_myo_) was inserted into the model using the measured volume ratios (*R*_1_, *R*_2_, and *R*_3_) based on the histological examinations. A few studies have presented three-compartment models using fluorescence data [11,28]. These involved a pharmacokinetic model for chloro-aluminum sulfonated phthalocyanine in an implanted hamster cheek pouch carcinoma tumor model [28]. However, only the fluorescence in the tumor and normal tissue compartments was measured. In contrast, we measured the myocardial fluorescence considering the origin of the fluorescence from plasma, interstitial space, and cell compartments. Another three-compartment pharmacokinetic model used radioisotope to estimate the interstitial concentration [11]. In contrast, we measured myocardial florescence, which is an easier and safer approach compared with the reported compartment model method using radioisotope. We believe our methodology is the first to use measured changes in talaporfin sodium plasma concentration and myocardial fluorescence that considers the origin of the fluorescence from the compartments with the measured volume ratio based on histological examinations.

### 4.2. Application of the Three-Compartment Model

As shown in Figure 5, the estimated concentration peak of talaporfin sodium in the interstitial compartment was 17 μg/mL at 8 min after injection, which was equal to 29% of the initial talaporfin sodium concentration in the plasma compartment. In terms of the interstitial PR scheme, including for infectious disease or myocardial ablation [1,2,3,4,7], this peak of interstitial concentration might be useful for enhancing the interaction efficacy. After reaching its peak, the estimated interstitial concentration tended to gradually decrease in line with the plasma concentration, and approximately 1 h after injection, the concentrations in the plasma and interstitial compartments were almost the same. This indicated that the distribution of talaporfin sodium reverted to a two-compartment model after one hour. We confirmed the advantage of the three-compartment model for characterizing the concentrations for the first hour after injection compared with a two-compartment model. Using the constructed three-compartment model, the optimum time for obtaining maximal interaction efficacy for an interstitial PR scheme could be estimated. To obtain the pharmacokinetics of the interstitial space in a certain tissue, a tissue fluorescence time history needs to be determined in the way we propose.

### 4.3. Limitations

Firstly, although optimization of the three-compartment model was confirmed, the accuracy of the optimized rate constants was not evaluated. The accuracy of the optimized kinetic rate constants can be confirmed by performing the study in at least 3–5 animals, if not more. Secondly, we used the compartment volumes of glucose as the functional compartment volumes of talaporfin sodium (sum of *V*_2_ and *V*_3_). These values for glucose are not perfectly applicable to those of talaporfin sodium. The pharmacokinetic properties, such as absorption, distribution, metabolism, and excretion process, could essentially be different between glucose and talaporfin sodium. Based on our model construction policy regarding the optimized parameters, we wanted to describe the functional volumes of each compartment for talaporfin sodium under the assumption that the pharmacokinetics for talaporfin sodium and glucose would be similar because both materials are water-soluble and close in molecular weight for canine model. Thirdly, we were only able to provide a few data points for the measured myocardial fluorescence because it was hard to stably measure the fluorescence from the beating heart of the open-chested animal. Finally, we only produced one series of interstitial fluorescence dynamics in one canine. We did not use multiple animals or data sets. The use of only one animal reduces the significance and reproducibility of the methods proposed. 

## 5. Conclusions

We have described a methodology for constructing a three-compartment pharmacokinetic model to estimate myocardial interstitial concentration using measured changes in talaporfin sodium plasma concentration and changes in myocardial fluorescence that consider the origin of the fluorescence from the compartments with measured volume ratios based on histological examinations. We propose that this method can be applied to other tissues.

## Appendix A: How to Obtain a Conversion Constant (*R*_myo_)

A conversion constant (*R*_myo_) was determined using the myocardial measured fluorescence data (*M*_myo_) and *C*’_myo_. We assumed that the initial value of the calculated myocardial concentration [*C*_myo_(0)], which was obtained from Equation (6) using the determined *C*_1_(0): 59.01 μg/mL and *C*_2_(0), and *C*_3_(0): 0 μg/mL described in Section 2.4, was equal to the initial value of the myocardial measured concentration data [*C*′_myo_(0)]. *R*_myo_ was set to be 189 μg/(mL·counts) using *M*_myo_(0) = 0.025 counts and *C*_myo_(0) = 4.72 μg/mL.
